# Neural Correlates of Alerting and Orienting Impairment in Multiple Sclerosis Patients

**DOI:** 10.1371/journal.pone.0097226

**Published:** 2014-05-12

**Authors:** Manuel Vázquez-Marrufo, Alejandro Galvao-Carmona, Javier J. González-Rosa, Antonio R. Hidalgo-Muñoz, Mónica Borges, Juan Luis Ruiz-Peña, Guillermo Izquierdo

**Affiliations:** 1 Experimental Psychology Department, Faculty of Psychology, University of Seville, Seville, Spain; 2 Multiple Sclerosis Unit, Virgen Macarena Hospital, Seville, Spain; 3 Laboratory for Clinical Neuroscience, Centre of Biomedical Technology (CTB), Technical University of Madrid (UPM), Madrid, Spain; San Raffaele Scientific Institute, Italy

## Abstract

**Background:**

A considerable percentage of multiple sclerosis patients have attentional impairment, but understanding its neurophysiological basis remains a challenge. The Attention Network Test allows 3 attentional networks to be studied. Previous behavioural studies using this test have shown that the alerting network is impaired in multiple sclerosis. The aim of this study was to identify neurophysiological indexes of the attention impairment in relapsing-remitting multiple sclerosis patients using this test.

**Results:**

After general slowing had been removed in patients group to isolate the effects of each condition, some behavioral differences between them were obtained. About Contingent Negative Variation, a statistically significant decrement were found in the amplitude for Central and Spatial Cue Conditions for patient group (p<0.05). ANOVAs showed for the patient group a significant latency delay for P1 and N1 components (p<0.05) and a decrease of P3 amplitude for congruent and incongruent stimuli (p<0.01). With regard to correlation analysis, PASAT-3s and SDMT showed significant correlations with behavioral measures of the Attention Network Test (p<0.01) and an ERP parameter (CNV amplitude).

**Conclusions:**

Behavioral data are highly correlated with the neuropsychological scores and show that the alerting and orienting mechanisms in the patient group were impaired. Reduced amplitude for the Contingent Negative Variation in the patient group suggests that this component could be a physiological marker related to the alerting and orienting impairment in relapsing-remitting multiple sclerosis. P1 and N1 delayed latencies are evidence of the demyelination process that causes impairment in the first steps of the visual sensory processing. Lastly, P3 amplitude shows a general decrease for the pathological group probably indexing a more central impairment. These results suggest that the Attention Network Test give evidence of multiple levels of attention impairment, which could help in the assessment and treatment of relapsing-remitting multiple sclerosis patients.

## Introduction

Cognitive impairment is evident in up to 70% of patients with confirmed multiple sclerosis (MS) [1]. The most frequently impaired cognitive domains in MS patients are processing speed, memory and attention [2], [3], [4]. To understand the neural basis of the cognitive impairment in this pathology several techniques have been used [5], [6], [7]. Cognitive potentials have been measured during the performance of a particular task to correlate behavior and physiology [8], [9], [10], [11], [12].

A few years ago, a particular task, the Attention Network Test (ANT), was developed by Fan et al [13]. It permits 3 attentional networks to be studied in a brief experimental session. These networks - alerting, orienting and executive - have been proposed on the basis of many studies, and their anatomical and physiological properties have been defined [14].

The attentional mechanisms studied by ANT are as follows [13], [14]. First, a general preparatory state or the "arousal" level needed for rapid detection of expected stimulus is managed by the alerting network, and is associated with increased activity in the right frontal lobe and right parietal lobe. These regions receive noradrenergic projections (related to alertness) from the locus coeruleus. Second, the movement of the attentional focus is allowed by the orienting network. The brain areas involved are the posterior parietal cortex, the thalamic pulvinar nucleus, the superior colliculus and the frontal eye fields. The orienting network is associated with the cholinergic system. Last, the executive network is responsible for conflict resolution (stimulus or response), error detection and inhibitory control, which is associated with the activity of the Anterior Cingular Cortex (ACC) and the lateral prefrontal cortex. These regions contain a large number of dopamine receptors, suggesting that the dopamine system is involved in the executive network.

Although these attentional networks have a certain degree of independence between them [13], [14], [15], other studies have demonstrated that interaction exists between networks. For instance, some ANT studies on healthy subjects show an interdependence between the alerting and executive systems [16], [17].

Some studies combining ANT design and Event-Related Potentials (ERPs) have tried to clarify the neural correlates of attentional mechanisms involved in this test. Neuhaus et al. [18] looking at the modulations of N1 in the ANT found that the amplitude was modulated by the alerting and orienting networks. In particular, low amplitude was present for the no cue condition, bigger for the double cue condition, and highest for the spatial cue condition. They also analyzed the effect on the P3 component caused by the congruence variable, showing that the congruent stimuli had higher amplitude than the incongruent stimuli. They indicated that the difference could be related to response inhibition, although the contribution of the difficulty of the task cannot be ruled out. In children, with ADHD and a healthy control group were analyzed by Kratz et al. [19] for potential modulations in the Contingent Negative Variation (CNV) interval. They found an increase of the amplitude in the late phase of the CNV for the spatial cue condition with respect to the neutral cue condition in healthy controls. However, no analyses were for the no cue condition or the central cue condition. Missonnier et al. [20], in analyzing all the conditions, concluded that the CNV amplitude was related with the amount of information given for the cue. The CNV amplitude was larger for the more informative spatial cue (alerting and orienting, temporally and spatially informative) compared to the less informative central cue (alerting, temporally informative), and obviously in the absence of a cue. In line with this evidence, only one study has investigated the functional significance of CNV in different clinical forms of MS patients, using, however, a Posner cuing task. Gonzalez-Rosa et al. [9] found a reduction in amplitude in the initial phase of the CNV in their MS patients. This “early” CNV has usually been related to sensory processes associated with evaluating the information contained in the warning, and is functionally interpreted in terms of activation of an executive mechanism controlling orientation or attention to a stimulus [21], [22], [23]. This reduction of amplitude suggests a reduced or worse activation of orientation and preparation mechanisms in some MS patients following the presentation of a cue [9].

Using ANT and behavioral measures in MS patients, only 3 studies have demonstrated an impairment of the alerting network, but no effect on the orienting network [24], [25], [26]. Only one study has tried to identify the possible neural correlates related with the behavioral impairment in MS applying the ANT [24], however only structural MRI parameters were analyzed and no functional measurements were made.

Thus, this is the first study analyzing the ERP indexes (functional measures) and attentional deficits in a group of MS patients performing the ANT. Specifically, the study of the neurophysiological correlates in this test will allow the evaluation of the functioning of the three attentional networks and the alterations in patients with relapsing-remitting multiple sclerosis (RRMS). A better knowledge of the attentional impairment in this pathology and its neurophysiological basis could improve our capacity to assess and refine therapeutic strategies.

### Predictions

Considering that MS can cause a demyelination process and neuronal death, highly distributed attentional mechanisms can be impaired (alerting and orienting) and some neural correlates can be identified using ERP analysis [8], [9], [10], [11], [12]. In particular, we believe a reduction of the CNV amplitude can be expected for the patients group that reflects the impairment in alerting and orienting mechanism engaged by the cue. Moreover, a delay in the latency for early ERP components (P1 and N1) triggered by the target stimuli and a decrement for the amplitude in the P3 component related to impairment in more central cognitive processes are also expected. Finally, neuropsychological scores will probably show some degree of correlation with psychophysiological measures we have used (behavioral or ERP parameters), as described elsewhere [27], [28].

## Materials and Methods

### Ethics Statement

This study was carried out in compliance with the Helsinki Declaration. All participants signed informed consents before their inclusion and the protocol was approved by the ethics committee of the University of Seville (project code: PSI2010-16825).

### Patient Population and Study participants

Twenty-six patients with a definite diagnosis of relapsing-remitting multiple sclerosis (RRMS) according to the Poser criteria [29] were consecutively recruited in our MS Unit. The Expanded Disability Status Scale (EDSS) [30] was used in all the patients and the duration of disease in years was registered. Exclusion criteria included: forms of MS other than RRMS; <30 days free of clinical relapses; EDSS score over 6; presence of comorbid neurodegenerative or psychiatric disorders; history of alcohol or drug abuse; head trauma; vascular diseases and seizures; severe signs of depression; significant upper limb impairment; or visual acuity or field deficits. An equal number of healthy subjects were recruited for the study. Independent t-test and Chi-Square test were used to compare socio-demographic variables between groups.

### Neuropsychological Assessment

Before the ERP study, neuropsychological testing and depression assessment of the patients were given to well-trained psychologists blinded to the study goals. Cognitive functioning (attention, concentration and speed of information processing) was measured through the Paced Auditory Serial Addition Test, 3 seconds (PASAT-3s) [31], [32] and the Symbol Digit Modality Test (SDMT) [33], [34] and compared to the normative scores developed by Sepulcre et al. [35] Beck Depression Inventory (BDI-II) [36], [37] was used to assess symptoms of depression.

### Cognitive Task

**Figure 1:**
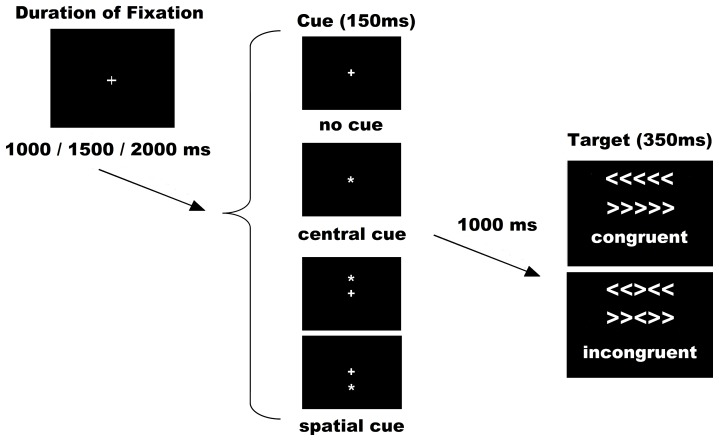
Experimental procedure. The possible combinations for sets of cues and targets were six: No cue congruent (NC-C), No cue incongruent (NC-I), Central cue congruent (CC-C), Central cue incongruent (CC-I), Spatial cue congruent (SC-C) and Spatial cue incongruent (SC-I).

The Attention Network Test (ANT) was used as per the original authors [13]. Stimuli consisted of a row of 5 horizontal white lines, with arrowheads pointing left or right, against a black background (see figure 1). There were 2 types of target stimuli: a congruent target (C), when the central arrow was flanked by other arrows pointing in the same direction, and an incongruent target (I), when the flanking arrows pointed in opposite directions. Target stimuli represented a total visual angle of 3.28 on the *x* axis and 0.41 on the *y* axis. The congruent and incongruent trials occurred in equal proportions. Under each condition (congruent or incongruent), half were pointing to the left and half to the right. The subject´s task was to indicate the direction of the central arrow by pressing the left button/arrow pointing to the left with the left thumb, or the right button/arrow pointing to the right with the right thumb.

The target was presented in one of two locations, either 0.86° above or below the fixation cross in the center of the display, the cross appearing in the center of the visual display throughout the entire experiment. To engage the alerting and orienting processes, a cue (an asterisk symbol) was shown before the appearance of target. There were 3 cue conditions: no cue (NC), central cue (at the fixation cross for alerting; CC), and spatial cue (at the target location for alerting plus orienting; SC). All cues occurred in the same proportions. Cues were displayed with a visual angle of 0.41^o^ on the x axis and 0.41^o^ on the y axis. In the NC condition, a black square the same size as the cue was shown (not visible to the subjects) to adapt all the timings for the different cue conditions and make them comparable for ERP analysis. As a result of the combination of target and cue conditions, the following 6 conditions were applied: No Cue Congruent (NC-C), No Cue Incongruent (NC-I); Central Cue Congruent (CC-C); Central Cue Incongruent (CC-I); Spatial Cue Congruent (SC-C) and Spatial Cue Incongruent (SC-I).

Some adaptations in the experimental procedure were made for our clinical group (see figure 1). The duration of the cue was 150ms before a fixed duration of 1,000ms. The target (with flankers) was then presented for 350ms. The time-window for participants' response was 1,000 ms after target onset and the duration between the offset of the target and the start of the next trial was variable (1,000ms; 1,500ms or 2,000ms).

The experiment consisted of 288 trials in 2 blocks of 144. All the trials (diverse cues and different possible targets) were randomly presented in both blocks. With respect to behavior analysis, as suggested by others [16], [17], we analyzed the interactions between conditions, but without subtractions (network effects) that could hide specific attentional mechanisms. Therefore, reaction time and accuracy were calculated for all conditions and averaged separately. Trials with an error were not included in the behavioral or ERP analysis. All the participants were instructed to respond as quickly and accurately as possible.

### EEG procedure

**Figure 2:**
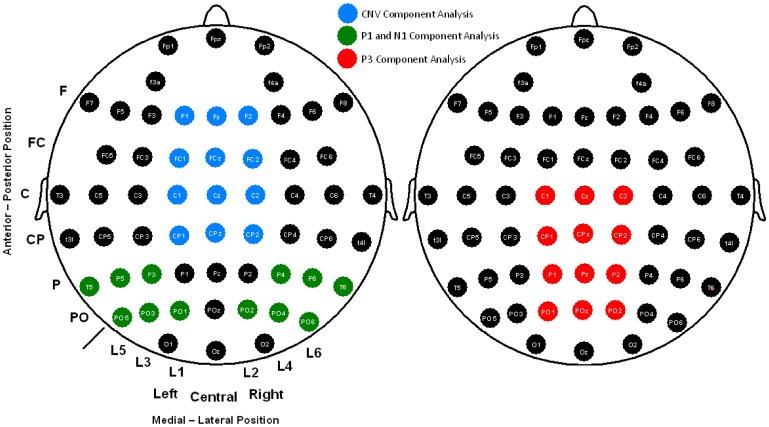
58 scalp electrodes recorded and sets of electrodes analyzed for each ERP (CNV, P1, N1 and P3) studied.

The electroencephalogram (EEG) was recorded from 58 scalp electrodes (Fp1, Fpz, Fp2, F3A, F4A, F7, F5, F3, F1, Fz, F2, F4, F6, F8, FC5, FC3, FC1, FCz, FC2, FC4, FC6, T3, C5, C3, C1, Cz, C2, C4, C6, T4, T3L, CP5, CP3, CP1, CPz, CP2, CP4, CP6, T4L, T5, P5, P3, P1, Pz, P2, P4, P6, T6, PO5, PO3, PO1, POz, PO2, PO4, PO6, O1, Oz, O2) (see figure 2), all of which were compared to an averaged reference. Vertical and horizontal electro-oculograms (VEOG and HEOG) were recorded. The electrode signals were amplified with BrainAmp amplifiers and digitally stored using Brain Vision Recorder software (Brain Products GmbH, Germany). The EEG signal was digitized at 500 Hz and filtered through the amplifier using a band-pass of 0.01–100 Hz, with the impedance below 5 kOhm during the experiment. Trials with a HEOG signal outside the ±75 µV range were rejected. To obtain a good and balanced signal-to-noise ratio between conditions, all the individual averages also comprised >45 artifact-free trials [38], [39].

CNV amplitude was analyzed for each cue condition in the time-window of 400 ms prior to the arrival of the target stimulus. As suggested by Duncan et al. (2009) [38], the latency and amplitude for P1 and N1 components were measured as follows: finding the electrode with the maximum amplitude, identifying the latency of this peak and then exporting the amplitude value at that latency for the rest of derivations included in the analysis. In the case of the P3 component, Pz electrode showed the maximum amplitude and had two peaks in some cases that were not recognizable in all subjects for latency analysis. Therefore, only amplitude analysis based in a range defined in the grand average (300–700 ms) was set for this component in both target conditions (congruent and incongruent). Derivations used to analyze latencies and/or amplitudes for all these components are depicted in figure 2.

### Statistical analysis

For behavioral analysis (reaction time and accuracy), a Mixed Repeated Measures ANOVA (MR-ANOVA) was used with the following factors and levels: Cue (No cue, Central cue and Spatial cue) x Congruence (Congruent and Incongruent) x Group (Patients and Controls). General slowing was analyzed as the result of the main factor “Group”. After this calculation, the Reaction Time (RT) data were corrected following the recommendation by Fernández-Duque and Black [40] to exclude differences between groups under all conditions caused by general slowing, and then an identical MR-ANOVA was used with the corrected data. The effects over specific experimental conditions could then be analyzed.

To analyze independently alerting and orienting attentional networks, CNV amplitude was analyzed by 3 MR-ANOVAs (one for each cuing condition) with the following factors (levels): Anteroposterior location factor (Frontal, Frontocentral, Central and Centro-parietal); Medial-lateral Position factor (Left, Central and Right location); and Group factor (Patients and Controls) (see figure 2).

To evaluate the interaction between attentional networks in the amplitude of P1 and N1 components between groups, 2 MR-ANOVAs were used (one for each component analyzed) with a 3×2×2×6×2 design: Cue factor (no cue, central cue and spatial cue), Congruence factor (congruent and incongruent), Anteroposterior location factor (Parietal and Parieto-occipital), Medial-Lateral Position factor (Line 5, Line 3, Line 1 (for the left hemisphere) and Line 2, Line 4, Line 6 (for the right hemisphere) and Group factor (Patients and Controls) (see figure 2).

P1 and N1 latency was analyzed using an MR-ANOVA for each component with a 3×2×2 design: Cue factor (no cue, central cue and spatial cue), Congruence factor (congruent and incongruent) and Group factor (Patients and Controls).

Amplitude modulations of the P3 component were analyzed with an MR-ANOVA with the following factors: Congruence factor (congruent and incongruent), Anteroposterior location factor (Central, Centro-parietal, Parietal and Parieto-occipital), Medial-Lateral Position factor (Left, Central and Right location) and Group factor (Patients and Controls) (see figure 2).

All variables were checked for normality using the Shapiro-Wilk test (p>0.05). A Greenhouse-Geisser correction for sphericity was applied and p ≤ 0.05 was considered significant. The Bonferroni correction was used in multiple comparisons post-hoc analysis.

A Spearman rank test was used to estimate the correlation between clinical data (i.e., EDSS, duration of the disease in years, and number of relapses) and behavioral parameters, neuropsychological scores and ERP measures.

Correlations between behavioral, neuropsychologicaļ and electrophysiological measures were computed using Pearsons's correlation coefficient. The global reaction time and the global percentage of correct responses were included as behavioral measures (ANT) in the correlation matrix, whereas the number of hits in the PASAT-3s, and the overall hit score in the SDMT, were used as neuropsychological measures. For ERP data, 6 variables were entered into the correlation analysis: the overall CNV, P1, N1 and P3 amplitudes, and the overall P1 and N1 latencies.

For correlation analyses, a statistical-significance of p<0.001 was determined after Bonferroni adjustment, 0.05 significance level divided by the total number (46) of multiple comparisons tested.

## Results

### Demographic and clinical data

MS patients and controls did not differ in socio-demographic variables. Both groups were equivalent with respect to sex, age, handedness, and education level (p>0.05). The EDSS had a mean value of 2.4±1.5. The mean year of the duration of the disease was 7.15±4.35 (see table 1).

**Table 1:** Demographic, neuropsychological and clinical data of experimental subjects.

	RRMS patients (n = 26)	Healthy controls (n = 26)
**Sex (m/f)**	10/16	15/11
**Age (years, mean±SD)**	34.42 (6)	30.31 (9.3)
**Handedness (left/right-handed)**	1/25	1/25
**Education (years, mean±SD)**	17 (4.92)	18.58 (3.87)
**Duration of disease (years, mean±SD)**	7.15 (4.35)	n.a
**EDSS (mean, range)**	2.4 (1–6)	n.a
**SDMT (mean±SD)**	44 (13.82)	n.a
**PASAT-3s (mean±SD)**	45.56 (14.34)	n.a
**BDI (II) (mean±SD)**	7.44 (6.3)	n.a

Key: RRMS: relapsing-remitting multiple sclerosis; m: male; f: female; EDSS: Expanded Disability Status Scale; SDMT: Symbol Digit Modality Test; PASAT-3s: Paced Auditory Serial Addition Test-3 seconds; BDI (II): Beck Depression Inventory-II; SD: standard deviation.

### Neuropsychological measures

About neuropsychological testing, the number of hits in the PASAT-3s and the overall hit rate in the SDMT were registered for all patients. Only the SDMT showed values of 2 SD under the cut-off scores (considering the normative scores from Sepulcre et al [35]), indicating an attentional impairment in our sample of relapsing-remitting MS patients (SDMT score  = 44±13.82). Lastly, BDI-II values indicated no severe signs of depression in this sample of patients (BDI-II score  = 7.44±6.3) (see table 1).

### Behavioral

Behavioral responses were slower in the patient group than the controls under all conditions (F1, 50 = 26.64; p<0.001; η^2^ = 0.35) (see table 2). After the general slowing had been removed in the MS group to isolate the effects of each condition [40], a statistically significant result was found for the interaction “Cue x Congruence x Group” (F2, 100 = 6; p = 0.005; η^2^ = 0.1) (table 2 gives for mean values under each condition). After post-hoc comparisons, all corrected values for all conditions differed between the groups except in No Cue-Congruent (p = 1.00) (see figure 1). Under some conditions (Center Cue-Congruent, Spatial Cue-Congruent and Spatial Cue-Incongruent), the difference was a lower corrected value in the control subjects, which means a better relative performance in this group for these conditions compared to patients. In others (No Cue-Incongruent and Center Cue-Incongruent), the patients had a lower value than the controls, which means a better relative performance into this group than in the control group for these conditions (note that a lower corrected value after general slowing correction represents a shorter reaction time than the average for each group). Regarding the accuracy of the responses, MR-ANOVA showed no significant differences for any of the main factors or their interactions between the 2 groups.

**Table 2:** ANT behavioral results.

Conditions	Correction not applied		Correction applied		P-value (post-hoc)
	RRMS (mean±SD)	Controls (mean±SD)	RRMS (mean±SD)	Controls (mean±SD)	
**NC-C**	592±93	485±69	0.97±0.04	0.97±0.04	1.000
**NC-I**	689±78	580±76	1.14±0.05	1.16±0.03	<0.001
**CC-C**	561±96	450±73	0.92±0.05	0.90±0.04	<0.001
**CC-I**	669±78	562±76	1.10±0.03	1.12±0.03	<0.001
**SC-C**	516±77	420±62	0.85 ±0.03	0.84±0.03	<0.001
**SC-I**	623±86	507±66	1.03±0.05	1.01±0.04	<0.001
**Mean RT**	607±80	500±68	n.a	n.a	<0.001 (*)

* The p-value refers to the “Group” factor before correction for general slowing had been applied (see text for details). Abbreviations: RRMS: relapsing-remitting multiple sclerosis; SD: standard deviation, RT: Reaction Time, NC-C: No cue congruent, NC-I: No cue incongruent, CC-C: Central cue congruent, CC-I: Central cue incongruent, SC-C: Spatial cue congruent and SC-I: Spatial cue incongruent.

### ERPs

#### Contingent Negative Variation

**Figure 3:**
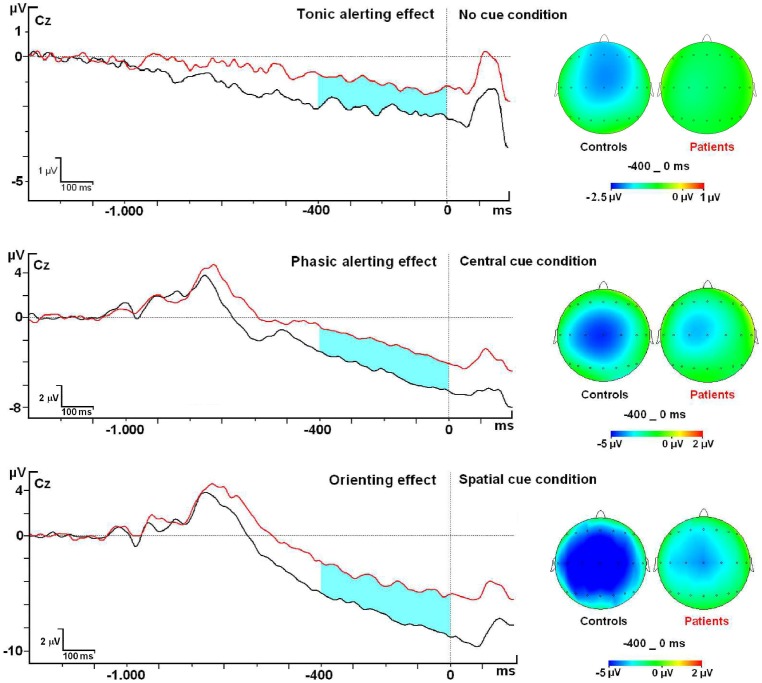
Contingent Negative Variation modulations at Cz electrode and topographic maps.

Controls and patients showed the maximum amplitude value for the Contingent Negative Variation (CNV) in the Cz and FcZ electrodes for all cue conditions. Amplitude analysis of modulations in the CNV related to the Cue Conditions showed no statistical difference between groups in the No Cue Condition (F1, 50 = 1.89; p = 0.17; η^2^ = 0.04) (Mean Control group: −1.69 µV±0.47; Mean Patient group: −0.99 µV±0.22) (see figure 3). However, analysis of modulations in the CNV related to the Central Cue condition between groups showed a statistically significant difference for the GROUP factor (F1, 50 = 6.1; p = 0.01; η^2^ = 0.11) (Mean Control group: −3.38 µV±1.05; Mean Patient group: −1.89 µV±0.8). Lastly, CNV amplitude in the Spatial Cue condition also showed a statistically significant difference between the groups (F1, 50 = 4.58; p = 0.03; η^2^ = 0.08) (Mean Control group: −5.18 µV±1.38; Mean Patient group: −3.51 µV±0.8).

#### The target P1 and N1 components

Both groups showed the maximum amplitude value for the P1 and N1 components in the PO5 and PO6 electrodes for all cue x target conditions. Table 3 summarizes latency values of the P1 and N1 component analyzed for each condition. MR-ANOVA showed a marginal effect in the P1 latency by GROUP factor. (F1, 50 = 4.012; p = 0.05; η^2^ = 0.07) (Mean Control group: 112 ms±20; Mean Patient group: 121 ms±23). With regard to the N1 component, MR-ANOVA showed an effect in the N1 latency by GROUP factor (F1, 50 = 6.659; p = 0.01; η^2^ = 0.12) (Mean Control group: 176 ms±16; Mean Patient group: 186ms±23). No significant cue, congruence or interactions of cue x congruence factors were found in any of the 2 components. Regarding the amplitude, no differences were found between groups (p>0.05) for the P1 and N1 components in any condition.

**Table 3:** Relevant results of latency and amplitude values found in this study.

	RRMS patients (n = 26)	Healthy controls (n = 26)	P-value
**CNV-NC amplitude (mean, SD)**	−0.99 µV±0.22	−1.69 µV±0.47	p = 0.17
**CNV-CC amplitude (mean, SD)**	−1.89 µV±0.8	−3.38 µV±1.05	p = 0.01
**CNV-SC amplitude (mean, SD)**	−3.51 µV±0.8	−5.18 µV±1.38	p = 0.03
**P1 latency (mean, SD)**	121ms±23	112ms±20	p = 0.05
**N1 latency (mean, SD)**	186ms±23	176ms±16	p = 0.01
**P3 amplitude (C+I) (mean, SD)**	3.06 µV±1.15	4.84 µV±1.33	p = 0.002

Amplitude values shown for CNV and P3 represents the mean value of the electrodes analyzed in each component and condition studied. Latency values shown the mean value at the peak of the six conditions analyzed for P1 and N1 components. Abbreviations: RRMS: relapsing-remitting multiple sclerosis; CNV: Contingent Negative Variation, NC: No cue condition, CC: Central cue condition, SC: Spatial cue condition, C: Congruent condition, I: Incongruent condition, SD: Standard deviation, ms: milliseconds and µV: microvolts.

#### The target P3 component

**Figure 4:**
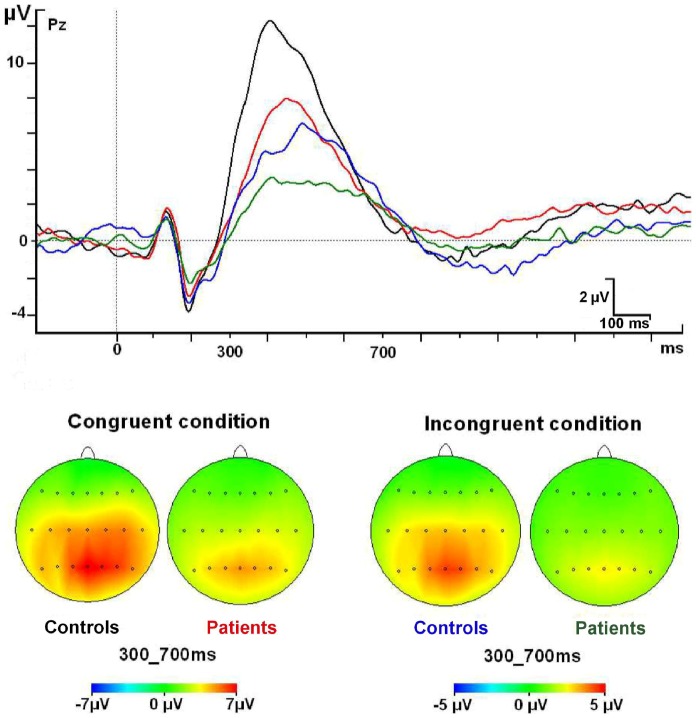
P3 component modulations at Pz electrode and topographic maps.

The maximum amplitude for the P3 component in both groups was found in parietal regions (Pz) for target conditions. There was a significant main effect for the GROUP factor (F1, 50 = 10.526; p = 0.002; η^2^ = 0.14), which indicates a decrease of the P3 amplitude in both conditions (congruent and incongruent) for the MS group. No other significant interactions were found (figure 4 and table 3).

#### Correlation analysis

**Figure 5:**
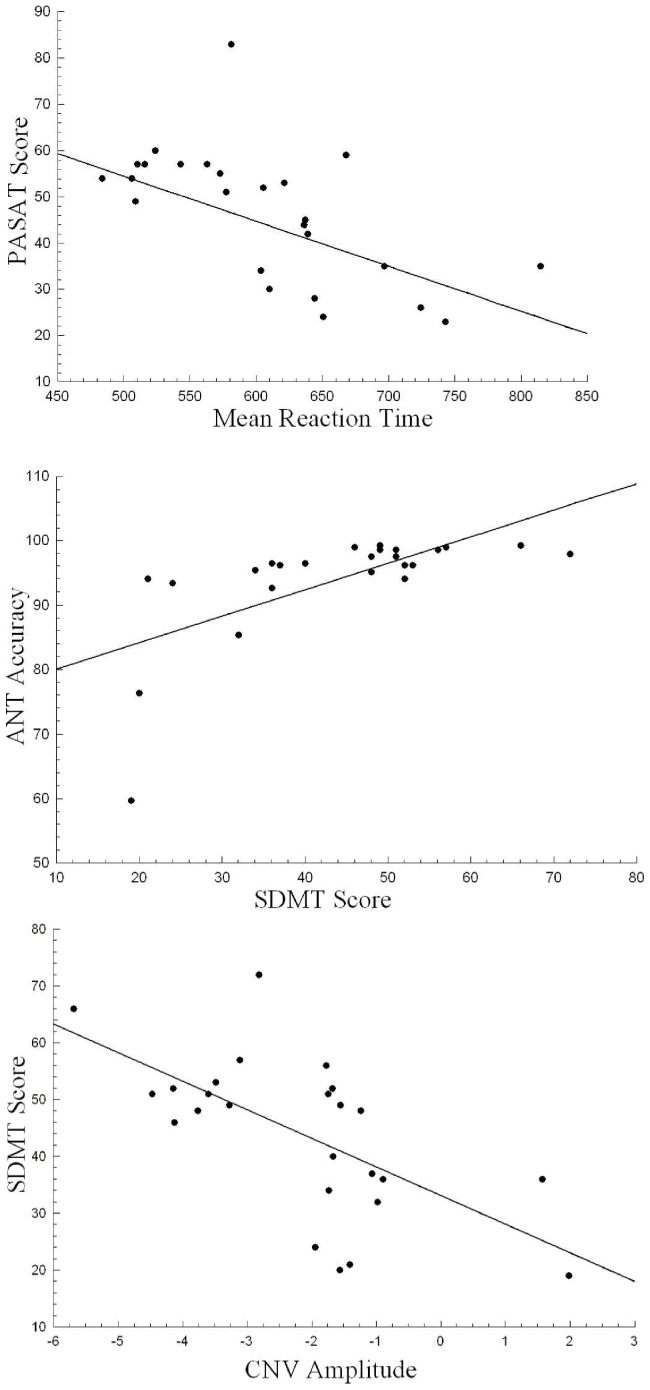
Correlation analyses. Neuropsychological score (PASAT-3s) and mean reaction time (upper panel); Accuracy in the Attention Network Test and SDMT score (middle panel), and CNV amplitude and SDMT score (lower panel).

EDSS, duration of disease in years and number of relapses did not correlate significantly with any of the behavioral, neuropsychological or ERP measures considered in this study. PASAT-3s was negatively correlated (r: −0.624, p = 0.001) with the Reaction Time (better performance in the neuropsychological test, lower reaction time). There was a positive correlation of accuracy of the responses with the SDMT (r: 0.654, p<0.001) (a better accuracy in the behavioral responses, a better score in the SDMT test). Lastly, the SDMT score and the overall CNV amplitude were negatively correlated (r: −0.635, p = 0.001) (better performance in the neuropsychological test, higher negative amplitude value) (see figure 5). After Bonferroni correction for multiple testing, no further significant correlations were detected between the neuropsychological scores and the ERP parameters.

## Discussion

A general slowing in reaction time for the RRMS group is seen here, as in previous studies [41], [42]. However, to isolate specific effects on the attentional mechanisms, a correction suggested by Fernández-Duque and Black [40] was applied. In particular, with the corrected values, the No Cue-Congruent condition did not differ between the 2 groups, but the No Cue-Incongruent performance was relatively better in the patient group than the controls. This result seems to demonstrate less interference between alerting and executive network in the patient group than the control group, probably because at some level the alerting system is impaired. This supports previous studies that described an alerting impairment and its interaction with the executive network in MS patients [24], [25], [26]. However, this effect is not strictly related to that considered by Fan et al. [13] In this case, we refer to a tonic alerting effect that is present even in the absence of a warning cue, i.e. the subject's basic level of alertness during the experimental session that is independent of the task. The CNV amplitude found in the No cue condition for both groups supports the idea that, even in here, this task elicits some level of alertness in subjects (see figure 3). However, no statistical differences between groups were found for this condition for the CNV. Therefore, no clear neurophysiological index was identified that related to this impairment of the tonic alerting effect, perhaps because it is a very subtle brain activity.

For Central cue conditions, some kind of impairment is also evident in the RRMS patients because no benefits accrued in the Central Cue-Congruent condition and no cost was found in the Central Cue-Incongruent condition. This suggests that a phasic alerting initialized by the Central cue is not operating properly, which could provoke impaired processing of upcoming stimuli. Others have proposed that a general preparation of motor systems occurs following a warning cue without spatial information, which is related to the CNV [9], [43], [44], [45]. Our data suggests that the decreased amplitude of the CNV in the RRMS patients indicates some impairment in the mechanisms in central cuing. This is the first time that modulation of this component in RRMS patients performing this paradigm has been described.

With regard to the orienting effect, the control group benefited more than the patient group for both conditions (Spatial Cue-Congruent and Spatial Cue-Incongruent). A similar result has been described in MS patients using a different attentional task, suggesting a compromised attentional mechanism in cue-orientation processes emerging in different stages of the disease [9], but this is the first time it has been recorded in the ANT paradigm for RRMS patients. A possible reason for the absence of orienting impairment in previous behavioral studies using this paradigm [24], [25], [26] is the difference in the SOA in the CNV interval. Previous studies employed a SOA between the cue and target onset of 400 ms, whereas ours was extended to 1150 ms to permit better involvement of the orienting network for both experimental groups. According to previous studies, CNV in spatial cuing is related to the preparation of sensory and motor areas for the subsequent stimulus [21], [22], [23]. Our data might be interpreted as a lower preactivation in the patient group and hence a poorer orienting behavioral response.

In the early event-related potentials, target P1 and N1 components showed delayed latency in all the conditions for the MS group. This reflects specific impairment in the first steps of the visual sensory processing, as noted in other studies as the result of the demyelination process [8], [46], and no atrophy as no modulations were found for the amplitude in both components. However, other studies have shown modulations in the amplitude of early components that suggest possible heterogeneity in the impairment provoked by this pathology [9], [11].

Lastly, the P3 component showed a statistical significant decrease in its amplitude for both congruent and incongruent conditions in the RRMS group. Many hypotheses have been posited to interpret decrements in the P3 amplitude. For instance, Kratz et al. [19] mentioned that a lower amplitude for the P3 component in ADHD children showed less attentional resources for this group. However, it is usually found in healthy subjects that the P3 component is smaller in the incongruent P3 compared to the congruent one when the Attention Network Test is used [47], [48]. A possible interpretation for this decrease is in fact the other way round. The P3 component, built by several mechanisms (evaluation of the stimuli, task relevancy, and so on) could show dispersion on time of these mechanisms when the difficulty is higher and more synchronization of these mechanisms when the task demands are lower. In the first case, bigger attentional demands lead to lower amplitude in the P3 and vice versa for the congruent condition (less demanding). On this reasoning, smaller P3 amplitude in the ADHD subjects could indeed reflect that these patients are more challenged by the task that causes smaller P3 amplitude, with something similar occurring with our sample of relapsing-remitting MS patients. We seem to find that the attentional demand is higher for the patients in both conditions (congruent and incongruent). However, it might be affirmed that the executive function could to some degree be impaired in MS patients as the incongruent condition shows less amplitude in this group. However, more mechanisms must be impaired as congruent condition shows a similar decrement. More research is needed to clarify all the reasons for this behavior in the P3 component for MS patients.

In the correlation analysis, neither the EDSS nor the duration of disease was strongly related to any of the behavioral and ERP measures. For the EDSS, previous studies reported lower correlations between EDSS and cognitive measures [49], [50], suggesting that the EDSS score (comprising diverse scores, not exclusively cognitive) is not a good predictor of cognitive disability. The duration of the disease has been related in previous studies to the conflict effect [25]; but, as we have pointed out, network effects (for instance, conflict or executive network as subtraction of congruent versus incongruent) were not considered in our present study as other authors recommend caution with these kind of subtractions [16], [17].

The neuropsychological measure, PASAT-3s, correlated inversely with reaction time, meaning that a better performance in this neuropsychological test corresponds to a shorter reaction time. Only SDMT correlated significantly with accuracy, better performance in the SDMT, greater accuracy percentage for the subject. All these correlations between neuropsychological measures and behavior suggest that this kind of test (ANT) could be complementary in studying attentional impairment. Behavioral measures are beneficial in computerized cognitive tests because of their precision in the time-scale and the possibility of studying specific mechanisms, which in the case of the neuropsychological tests are all included. This makes it difficult to identify the specific mechanism responsible for the attentional impairment [50], for instance, PASAT-3s is sensitive to general slowing, sustained and divided attention or working memory impairments.

The significant correlation between an ERP measure (CNV amplitude) and the SDMT score shows that some mechanisms involved in the performance of the neuropsychological testing are represented in the CNV, as has been proposed elsewhere for other ERP components (i.e. the P3 component) [11]. However, the lack of other correlations with PASAT and SDMT and the rest of the ERP components (P1, N1, etc.) indicate the difficulty of a simple interpretation of these correlations between neuropsychology scores and ERP measures. Further correlation analysis in cognitive paradigms and with different ERP components are needed to clarify this topic.

## Conclusions

In summary, MS patients show multiple attentional impairments. Both orienting and phasic alerting deficits seem to be related to a decrease in CNV amplitude. In the case of the spatial condition, this drop could be associated with a deficit in the preactivation of the sensory and motor systems to process new stimulus in new locations. In the case of the Central Cue condition, the decreased amplitude of CNV could be related to some impairment in the necessary mechanisms for a general preactivation when a warning cue is presented. The delayed latency for target P1 and N1 component shows that relapsing-remitting MS patients are impaired in the first steps of the visual sensory processing. Regarding the P3 component, reduced amplitude for the incongruent in the patient group could represent impairment for the executive network for these patients. However, modulations in P3 may not exclusively be caused for this reason, and other mechanisms could be involved. In the correlation analysis, none of the behavioral measures were related to the clinical variables, but all correlated strongly with the neuropsychological tests (PASAT-3s and SDMT), and CNV also showed a specifically high correlation with the SDMT. All these results suggest that attentional impairment in MS patients is a complex entity requiring multiple approaches for better assessment.
